# The Effect of Midsole Thickness on Running Economy, Spatiotemporal Values and Perceptions of Comfort and Exertion in Well-trained Runners: A Randomized, Cross-over Trial

**DOI:** 10.1186/s40798-025-00911-z

**Published:** 2025-10-01

**Authors:** Gian-Andri Baumann, Kai Biedermann, Enea Item, Christina M. Spengler, Fernando Gabe Beltrami

**Affiliations:** Exercise Physiology Lab, Institute of Human Movement Sciences and Sport, ETH Zurich, Exercise Physiology Lab, Gloriastrasse 37/39, 8092 Zurich, Switzerland

**Keywords:** Marathon, Super shoes, Energy expenditure, Performance

## Abstract

**Background:**

Distance running has been revolutionised by the introduction of modern running shoes, named advanced footwear technology (AFT). AFT shoes commonly implement carbon fibre plates to increase longitudinal bending stiffness as well as highly responsive midsole foams, which increase energy return. In 2022, World Athletics stipulated that midsoles in competition road running shoes cannot exceed 40 mm in thickness, but this limit seems arbitrary. Therefore, this study aimed to understand the effect of midsole thicknesses in AFT shoes at (40 mm) and above (50 mm) the current regulation on running economy (RE), perceived comfort and exertion, and spatiotemporal variables during treadmill and overground (400-m track) running, while comparing values to those of an entry level running shoe (EL).

**Results:**

On the treadmill, RE with the 40 mm shoes improved by 2.4 ± 1.1% compared with the EL shoes (56.0 ± 4.3 ml･kg^− 1^･min^− 1^ vs. 57.4 ± 4.2 ml･kg^− 1･^min^− 1^, *P* < 0.001) and improved further with the 50 mm shoes (55.7 ± 4.3 ml･kg^− 1･^min^− 1^, *P* = 0.042, -0.6 ± 1.2% vs. 40 mm). A similar pattern was seen during overground running, but no correlation was detected between the magnitude of differences for each pair of running shoes and running surface (*P* > 0.36). Both AFT models showed lower perceived exertion compared with the EL shoes, but no differences were detected between the AFT models. The 40 mm shoes were seen as more comfortable than both the 50 mm- and EL shoes on the treadmill as well as on the track. No differences were detected between any of the shoes with respect to ground contact time, step frequency, or step length.

**Conclusion:**

Increasing midsole thickness beyond current regulations can further improve RE, although it remains to be seen whether such differences would translate into performance. The 40-mm limit for midsole thickness imposed by World Athletics for road running shoes does not represent an upper limit beyond which no further gains can be obtained and therefore could be limiting the role of footwear in performance breakthroughs.

**Supplementary Information:**

The online version contains supplementary material available at 10.1186/s40798-025-00911-z.

## Key points

World Athletics imposes a 40-mm upper limit in shoe midsole thickness or stack height of road running shoes, but no reasoning is given for this criterion.

We demonstrate that shoes exceeding current limits can improve running economy compared with shoes complying with current rules, despite increased shoe mass.

Although the magnitude of differences between shoes was similar in both tested surfaces, no correlation was detected between surfaces, pointing to the need for more field research in this topic.

**Supplementary Information:**

The online version contains supplementary material available at 10.1186/s40798-025-00911-z.

## Background

Modern running shoes, recently termed advanced footwear technology (AFT), have revolutionized long distance running by improving running economy (RE) compared with traditional running shoes [1], allowing a higher running speed to be achieved for a given oxygen uptake (V̇O_2_). The underlying mechanisms behind the RE improvements provided by AFT are not yet fully understood but likely include a combination of minimal shoe mass with high compliance (capacity to provide cushioning and store elastic energy) and high resilience (i.e. the ability to return this energy) [2–4].

If the midsole foam is too thin, it can potentially reach a state of compression where it becomes completely rigid, which is referred to as the “bottom-out” phenomenon. Furthermore, a thicker midsole can improve the spring capacity of a responsive foam and therefore improve energy return during running [2]. Increased midsole thickness also leads to a longer lower limb length, which has been shown to decrease O_2_ cost of transport across different animal species [5]. On the other hand, excessively increasing midsole thickness will increase shoe mass and ankle instability [6], possibly leading to discomfort, all of which might offset any gains seen in other attributes.

Following the improvements in several road running distance records by runners wearing the Nike Vaporfly 4%, which was shown to boost RE by approximately 4% compared with traditional running shoes [1], a call was made to regulate the midsole thickness (also called stack height) of AFT shoes [7], to prevent shoes from having an oversized role in long distance running. At the time, this was met with scepticism in the literature based on the lack of strong evidence for any given limit [6, 8]. Later, in January 2022 World Athletics introduced a 40-mm upper limit in maximal midsole thickness for shoes worn during road running events, although no physiological or biomechanical reason was given for this upper limit. Interestingly, a few months later, in October of 2022, Gustav Iden won the Ironman World Championship in Kona while breaking both the overall and marathon course records (the marathon mark remains the course record in 2025), finishing the running leg in 2:36:15 or 16.2 kmཥh^− 1^. At the occasion, he was wearing a pair of prototype shoes which had a midsole thickness of 50 mm. Soon after that event, World Triathlon decided that from the beginning of 2023 it would follow similar shoe rules as World Athletics.

Even though the prototype 50-mm shoes had undisputable success in the field, subsequent research does not support the notion the increasing midsole thickness beyond 40 mm brings metabolic advantages: a single peer-reviewed study to date has evaluated the effects of shoes bearing AFT characteristics with varying midsole thicknesses [9], reporting no differences between midsoles thicknesses ranging from 35 mm to 50 mm. Another recent report [10], not yet peer-reviewed, seems to nonetheless support these findings, expanding on a prior work by the same group with a reduced sample size of only five participants [11]. Although other studies on the topic used different shoes altogether [12], or added slats of foam directly to the treadmill belt [13], they also fail to show a benefit from midsoles exceeding 40 mm.

Despite the evidence against possible RE benefits from increasing midsole thickness beyond 40 mm, it should be considered that other components of shoe construction might influence RE beyond mass and energy return alone. Thus, this study set out to investigate the effects of midsole thickness beyond 40 mm using race ready samples identical to those worn by Gustav Iden in his record-setting race. To do so, we compared RE and perceptual variables in well-trained runners while wearing an AFT shoe compliant with World Athletic regulations (midsole thickness 40 mm) and a prototype version (midsole thickness 50 mm). To do so, we first sought to establish that the rule-compliant shoes indeed showed the expected effects from an AFT model and further compared it to the prototype shoe version. In addition, we sought to extend from the current literature by performing the tests on a motorized treadmill but also during overground running on an athletics track. Considering that a thicker midsole can increase the ability to store and release energy [2] and that a softer running shoe can potentially affect perception of comfort and exertion and thus RE [14, 15], we hypothesised that running shoes with a 50-mm midsole thickness would further improve RE and reduce perceived exertion during running compared with a race legal AFT model, despite the inherent increase in shoe mass.

## Methods

Twenty-one well-trained runners from local running clubs (male *n* = 20, age 28 ± 6 years, height 180 ± 5 cm, body mass 70.3 ± 5.2 kg, body fat 12.0 ± 3.5%, V̇O_2peak_ 63.6 ± 4.2 ml⋅kg^-1^⋅min^-1^, peak treadmill running speed 20.2 ± 0.8 kmཥh^-1^, all mean ± SD) wearing shoe sizes US 9, 10 or 11 participated in this study. All participants had completed a marathon in less than 2 h and 50 min or a half marathon in less than 1 h and 21 min. All participants provided written, informed consent to participate prior to the first visit. The study was approved by the Ethics Committee of the ETH Zurich (2023-N-116) and was carried out according to the ethical standards of the Declaration of Helsinki, except for registration in a public database.

### Study Design

Participants performed three experimental visits, each separated by at least 72 h. In the first visit, participants were familiarized with the testing equipment and shoes and underwent assessments of body composition, anthropometry and an incremental running test for determination of peak oxygen uptake (V̇O_2peak_). On visits 2 and 3, which were performed in randomized and balanced order between participants, the participants performed the indoor and outdoor running sessions. Each session consisted of four runs in each of three different shoes, as described below. The final five participants performed five sets in each shoe instead of four during the outdoor trial, but only the data from the first four sets was used in this manuscript, for consistency between participants. This was performed to address a separate question not pertinent to the data reported in this study. Participants were requested to avoid training in the 24 h prior to a visit, and to avoid strenuous training 48 h before each visit. On the day of testing, participants were required to ingest their usual pre-race meal, at least 60 min before the start of a testing session.

### Familiarisation and Incremental Test

During the first visit, participants signed the informed consent form, completed a questionnaire on their health status, current sleep and training situation, and underwent a dual X-ray absorptiometry scan (GE Healthcare, Madison, USA) to assess segmental body composition. In addition, the participants performed an incremental test on a motorised treadmill (Pulsar 3p, H/P/Cosmos, Germany) to determine their V̇O_2peak_ and peak treadmill running speed. Following the body composition scan, participants completed a 15-min warm-up on the treadmill at a self-selected speed with a 1% incline. As part of the familiarisation process, each of the three different footwear conditions was worn for five minutes during this period. For the incremental test, the incline remained constant at 1% throughout the test and the starting speed of 10 km·h^-1^ was the same for all participants. Each stage lasted 60 s, and the speed increased by 1 km·h^-1^ from one stage to the next. The test ended when the runner voluntarily stopped because of exhaustion. Participants breathed through a facemask that was connected to an ergospirometer (Quark, Cosmed, Rome, Italy). Gas exchange and heart rate data (HR, Polar H10, Polar, Kempele, Finland) were monitored throughout the incremental test. Prior to the running bouts, the ergospirometer was calibrated according to the manufacturer’s instructions, including the flow and gas concentration sensors.

### Indoor Protocol

The indoor test was performed on the same treadmill as the incremental test and used the same ergospirometer. Before the standardised warm-up, which consisted of a 5-min run at 14 km·h^-1^, The experimental running protocol included twelve 5-min runs at a speed of 16 km·h^-1^ and 1% inclination. To reduce the burden on the participants, the twelve runs were divided into four sets, each consisting of one run in each shoe. Rest between runs was 5 min, and 8 min between sets. The shoe order was randomised, balanced, and mirrored (e.g., A-B-C / C-B-A, repeated twice). Immediately after each run, the participants indicated their perceived comfort and exertion using a visual analogue scale (VAS) and a modified 1–10 Borg scale. The VAS featured a range from “not comfortable/no exertion” (point zero) on one end to “maximally comfortable/maximum exertion” on the other end. The participants received two separate sheets of paper, each with either the comfort or exertion scale printed on it. Gas exchange and HR were continuously monitored throughout the trial. Furthermore, the participants wore an accelerometer pod at waist level (± 16 g range, sampling at 1,024 Hz, Runeasi, Tienen, Belgium), which was used to collect spatiotemporal variables (see Data treatment and statistics for a list of variables).

### Outdoor Protocol

Following a standardised warm-up procedure, athletes ran on an outdoor 400 m athletic track for a total of three laps at a speed of 16 km· h^-1^, which is equivalent to 4 min and 30 s. The shoe order was kept the same for each participant for both the indoor and outdoor measurements. The twelve runs were divided in the same way as in the indoor session, and the same breaks were provided between the runs. To ensure that the runners maintained the correct pace, trained personnel stationed at the 0 and 200 m marks of the track provided pacing feedback. During the runs, participants breathed through a face mask connected to a portable ergospirometer (Metamax, Cortex, Leipzig, Germany), which was carried by the participant on a chest vest. In addition, HR and spatiotemporal variables were also assessed using the same devices as for indoor measurements. Perceived comfort and exertion were evaluated using the same VAS and Borg scale as indoors.

### Footwear

**Fig. 1:**
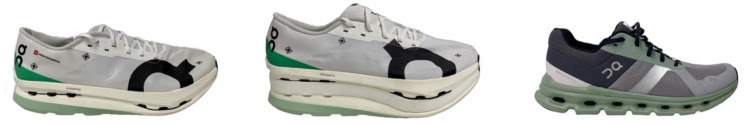
**Left** On Cloud Boom Echo 3.0 (40 mm), **Centre** On Cloud Boom Echo 3.0 Prototype (50 mm), **Right** On Cloud Runner (EL)

Three different types of footwear were used for this study, all supplied by On Running AG (Fig. 1). These were an entry-level running shoe (EL, On Cloud Runner, ethyl vinyl acetate midsole foam, weighting 300 g for a US 10 shoe, midsole thickness of 30 mm) and two carbon-plated modern running shoes with midsole thicknesses of 40 mm (40 mm, On Cloud Boom Echo 3.0, polyether block amide (PEBA) midsole foam, weighting 205 g for a US 10 shoe) and 50 mm (50 mm, On Cloud Boom Echo 3.0 prototype, PEBA midsole foam, weighting 250 g for a US 10 shoe), respectively. An additional 10 mm PEBA foam, and the inherent increased mass, was the only distinction between the 40 mm and 50 mm shoes. The shoes were not equalised for their mass.

### Mechanical Testing Protocol

Deformation, stiffness, resilience, and energy returned in the heel and forefoot of the three different shoes used were evaluated using a Zwick/Roell material testing machine (LTM Torsion, Zwick/Roell GmbH & Co. KG, Ulm, Germany). The shoe was placed in the centre of the piston drop line and secured with additional levers on each side. For the heel, it was placed in the centre of the heel cup, while for the forefoot, it was positioned 18 cm from the heel toward the toes in size 10.0 (only tested size). The protocol lasted for 60 cycles and was started by activating the piston, which unilaterally moved in an upward and downward motion at a frequency of 2 Hz, applying a set force of 1.5 kN for the heel protocol and 1.8 kN for the forefoot protocol. The tests were carried out both before the shoes were used in the study and after all the trials were completed.

### Data Treatment and Statistical Analysis

Data were collected throughout all individual runs, but only a two-minute window was used for further statistical analysis. More specifically, minutes 2:45 to 4:45 in the indoor protocol and 2:15 to 4:15 in the outdoor protocol. Data were exported in 5-s intervals, and values exceeding three standard deviations from the local mean were excluded as outliers. Energy expenditure was calculated [16]from gas exchange data and converted into Wཥkg^-1^ as a measure of metabolic power.

Spatiotemporal and kinetic variables were averaged for the last 90 s of each trial (excluding the final 10 s). This included step frequency, flight time, ground contact time, duty factor (defined as stance time divided by step time) impact magnitude and impact duration (defined as the time from foot strike until peak force is reached). Based thereon, other variables were calculated, including dynamic stability [17] – the proportion of hip movement in medio-lateral direction during landing as a proportion to the sum of triaxial movement – as well as vertical and leg stiffness [kN·m^-1^], the latter two calculated according to Morin [18]. For the values calculated directly by the Runeasi device, the within day typical error of measurement was calculated for each shoe using each run as individual replicates, expressed as a coefficient of variation as proposed by Hopkins [19].

Gas exchange, HR and spatiotemporal variables were compared between shoes by averaging the four replicates in each shoe condition using a one-way ANOVA with repeated measures. In cases where a significant main effect for the shoe was observed, pairwise comparisons were performed using the Holm-Sidak approach. Associations between different variables were tested using Pearson’s product moment correlation coefficients. To test whether the changes between shoes were different when tested during overground and treadmill running, the relative delta between shoes measured in both surfaces was compared using 2-tailed dependent t-tests. To test for a potential effect of familiarization in the perception of comfort, values for day 1 and day 2 (i.e., regardless of whether treadmill or overground running) were compared using a 2-tailed dependent t-test for each shoe. To test for potential changes in V̇O_2_ within day for a given shoe, the slope for V̇O_2_ against run (i.e., first, second, third and fourth run for a given shoe) was calculated for each participant. The slopes were then compared against zero using a uni-sample t-test. In both the latter analyses the multiple p-values were corrected to control the false discovery ratio (kept at 5%) using the method of Benjamini, Krieger and Yekutieli. All statistical testing was performed using Graphpad Prism (La Jolla, CA) version 10.4. Statistical significance was set at ⍺ < 0.05.

## Results

### Shoe Properties

The results of the mechanical tests (Table 1) showed that the 50 mm shoes exhibited approximately 61% higher compliance on the forefoot compared with the 40- mm and 116% higher compared with the EL shoes. This was evident from the deformation measurements, with the 50 mm shoes deforming 54.8% more compared with the 40 mm and more 111.2% more compared with the EL shoes. On the other hand, the resilience of the 50 mm shoes (82.2%) was slightly lower compared with that of the 40 mm shoes (85.1%) but higher than that of the EL shoes (74.5%). Overall, the 50 mm shoes could return 66% more mechanical energy compared with the 40 mm and almost three times more than the EL shoes. The heel analyses showed the same pattern (Table 1). The mechanical testing performed after the study finished (Table 1, performed with the most used size; US 10, 150 km of use) shows that although small variations were present the differences between shoes remained consistent.

**Table 1:** Mechanical properties of the different shoes used

		Heel			Forefoot		
		40 mm	50 mm	EL	40 mm	50 mm	EL
Shoe Mass (g)		205	250	300	205	250	300
Compliance (mm･N^-1^)	0 km	0.013	0.019	0.013	0.008	0.013	0.006
	150 km	0.013	0.019	0.012	0.009	0.013	0.006
Deformation (mm)	0 km	19.9	28.5	19.9	14.6	22.6	10.7
	150 km	20.0	29.2	17.3	15.8	23.4	10.6
Stiffness (N･mm^-1^)	0 km	74.7	52.0	78.0	121.1	78.9	166.7
	150 km	74.0	50.8	85.8	113.1	76.2	168.7
Returned Energy (J)	0 km	8.9	14.2	7.9	7.2	12.0	4.3
	150 km	8.7	13.5	7.2	7.3	12.5	4.0
Resilience (%)	0 km	85.5	85.2	75.7	85.1	82.2	74.5
	150 km	84.9	84.8	74.7	83.8	84.6	73.9

40 mm, AFT shoe with midsole thickness of 40 mm; 50 mm, AFT shoe with midsole thickness of 50 mm; EL, entry-level shoe

### Physiological Responses EL Vs. 40 mm

**Fig. 2:**
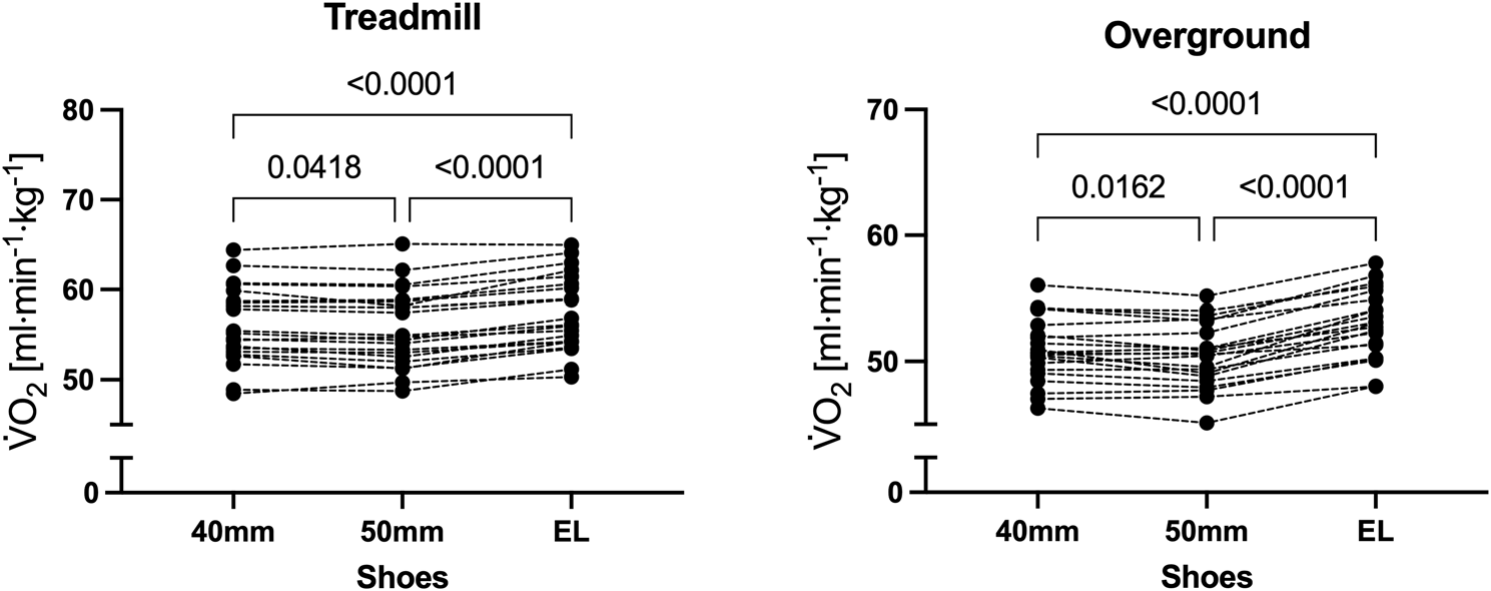
Individual data for V̇O_2_ while running on the treadmill (left) or overground (right) with three different shoes. 40 mm, AFT shoe with midsole thickness of 40 mm; 50 mm, AFT shoe with midsole thickness of 50 mm; EL, entry-level shoe. Pairwise comparisons were performed using the Holm-Sidak approach following a repeated-measures ANOVA

**Fig. 3:**
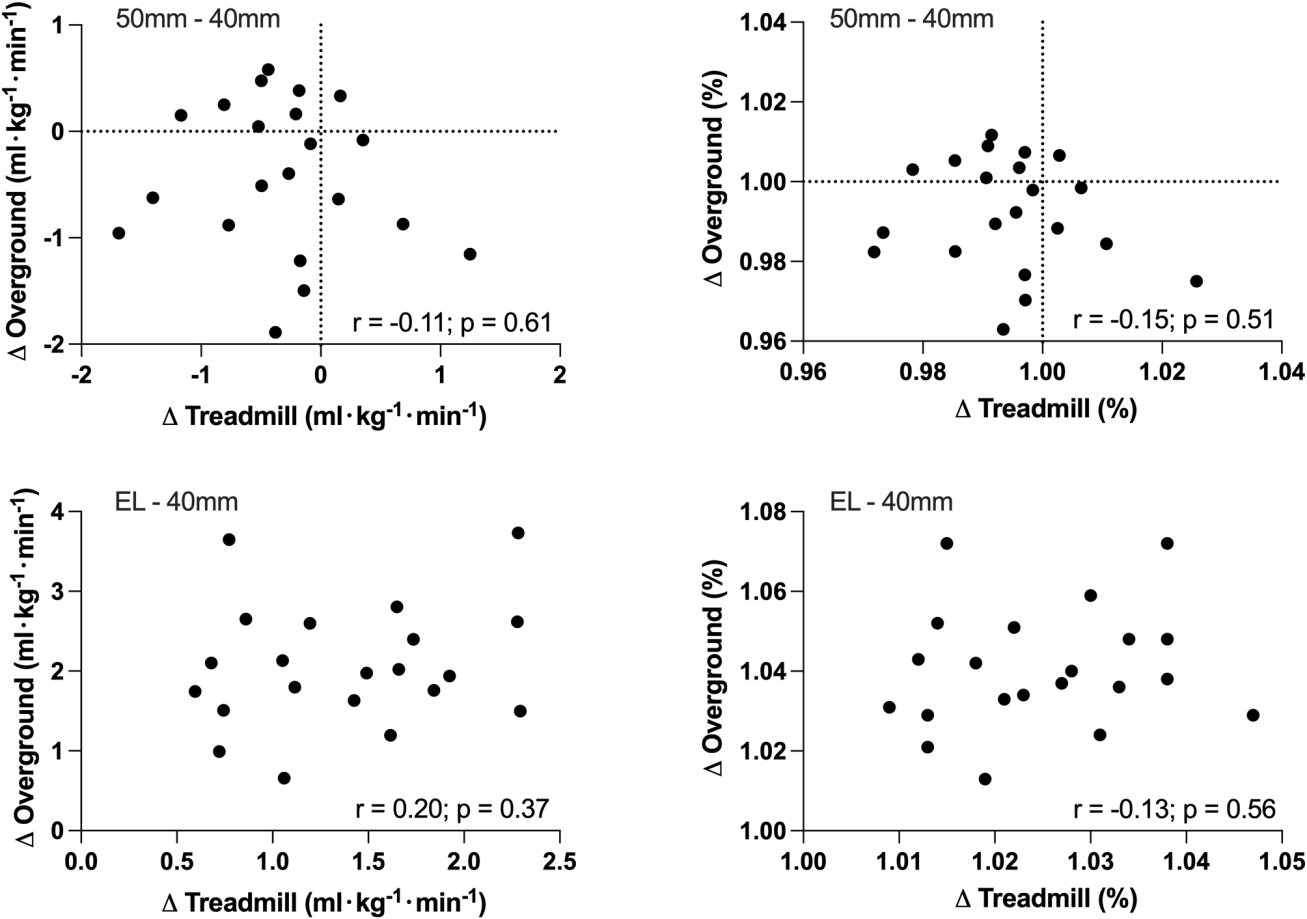
Correlation between the absolute (left panel) and relative (right panel) differences in RE between the 40 mm and 50 mm shoes (top) and between the EL and 40 mm shoes (bottom). R and p values are from a Pearson’s product moment correlation analysis. 40 mm, AFT shoe with midsole thickness of 40 mm; 50 mm, AFT shoe with midsole thickness of 50 mm; EL, entry-level shoe

Individual values for RE are presented in Fig. 2, while group means are available in Table 2 and individual numerical values for each run are provided as supplementary information. During treadmill testing, RE was 2.5 ± 1.1% superior when running with the 40 mm shoes compared with EL (-1.4 ± 0.6 mlཥkg^-1^ཥmin^-1^, *P* < 0.001). During overground running, RE was 3.9 ± 1.3% superior when running with the 40 mm shoes compared with EL (-2.0 ± 0.7 mlཥkg^-1^ཥmin^-1^, *P* < 0.001). The changes in RE between shoe conditions were larger during overground running compared with treadmill running (1.4 ± 1.5% points higher for overground compared with treadmill running, *P* < 0.001). Measures of metabolic power followed a similar trend (Table 2), but differences between shoes were slightly larger in magnitude (i.e. -2.8 + 1.0% during treadmill running and − 4.1 ± 1.6% during overground running), as RER was lower for the shoes with improved RE (Table 2). No relationship between the shoe models changes assessed during overground and treadmill testing could be detected (Fig. 3), although all participants showed improved RE with the 40 mm shoes.

**Table 2:** Physiological and perceptual responses to the different shoes

	Treadmill			Overground		
	40 mm	50 mm	EL	40 mm	50 mm	EL
V̇O_2_ (ml･kg^-1･^min^-1^)	56.0 ± 4.3£££	55.7 ± 4.3£££#	57.4 ± 4.2	50.9 ± 2.5£££	50.5 ± 2.5£££#	53.0 ± 2.7
Met. Power (W･kg^-1^)	19.2 ± 1.4£££	19.1 ± 1.3£££#	19.7 ± 1.4	17.7 ± 0.9£££	17.6 ± 0.9£££#	18.5 ± 1
RER	0.92 ± 0.05£££	0.92 ± 0.05£££	0.95 ± 0.05	0.94 ± 0.03£££	0.94 ± 0.03£££#	0.96 ± 0.03
HR (bpm)	174.3 ± 10£££	173.6 ± 9.8£££##	176.5 ± 9.6	167.9 ± 10.4£££	166.9 ± 10.1£££##	170.8 ± 10.1
V̇_E_ (L)	122.7 ± 19.7£££	121.5 ± 15.8£££	128.1 ± 18.5	112.2 ± 18.2£££	111.1 ± 16.5£££	120.1 ± 17.7
V_T_ (L)	2.6 ± 0.4	2.6 ± 0.4££	2.7 ± 0.4	2.5 ± 0.4££	2.5 ± 0.4£££	2.7 ± 0.4
f_B_ (breathsཥmin^-1^)	47.0 ± 7.5	47.4 ± 7.2	48.1 ± 7.6	44.9 ± 7.9	44.8 ± 8££	46.0 ± 8.4
Exertion (Borg, in units)	5.1 ± 1.2£££	5.0 ± 1.2£££	5.7 ± 1.1	4.2 ± 1.1£££	4.2 ± 1.2£££	4.8 ± 1.2
Exertion (VAS, in mm)	5.8 ± 1.2£££	5.6 ± 1.3£££	6.6 ± 1.1	5.0 ± 1.3£££	5.0 ± 1.2£££	5.6 ± 1.4
Comfort (VAS, in mm)	7.4 ± 1.5£££	6.5 ± 1.7^##^	5.8 ± 1.9	7.1 ± 1.5££	5.9 ± 2##	5.7 ± 2.1

Data are mean ± standard deviation. V̇O_2_, oxygen uptake; Met. Power, metabolic power; RER, respiratory exchange ration; HR, heart rate (in beats per minute, bpm); V̇_E_, minute ventilation; V_T_, tidal volume; f_B_, breathing frequency, VAS, visual analogue scale. 40 mm, AFT shoe with midsole thickness of 40 mm; 50 mm, AFT shoe with midsole thickness of 50 mm; EL, entry-level shoe. £ different from EL; # different from 40 mm. The number of symbols denote £, *P* < 0.05; ££, *P* < 0.01; £££, *P* < 0.001

### Physiological Variables 40 mm Vs. 50 mm

During treadmill testing, RE was 0.6 ± 1.2% superior when running with the 50 mm shoes compared with 40 mm (-0.3 ± 0.7 mlཥkg^-1^ཥmin^-1^, *P* = 0.042). During overground running, RE was 0.7 ± 1.3% superior when running with the 50 mm shoes compared with 40 mm (-0.4 ± 0.7 mlཥkg^-1^ཥmin^-1^, *P* = 0.023). No differences could be detected in the changes between shoes assessed during overground and treadmill running (0.1 ± 1.9% points higher for overground compared with treadmill running, *P* = 0.734). Measures of metabolic power followed a similar trend (i.e. -0.6 + 1.3% during treadmill running and − 0.8 ± 1.4% during overground running), as RER was very similar between shoes despite the small changes RE (Table 2).

Although the magnitude of the differences in RE between shoes were consistent at a group level in both testing conditions, the differences between shoes measured during treadmill running at an individual level were not associated with those measured during overground running, irrespective of whether absolute or relative differences in RE were used (Fig. 3). Only 10 out of the twenty-one participants showed a consistent response (i.e. either improved or worsened RE for a given shoe) in both surfaces.

### Other Physiological Responses

When compared within day, running V̇O_2_ slopes during treadmill were significantly non-zero for the 40 mm (-0.43 mlཥkg^-1^ཥmin^-1^ཥrun^-1^, *P* = 0.007), 50 mm (-0.46 mlཥkg^-1^ཥmin^-1^ཥrun^-1^, *P* = 0.005) and EL (-0.57 mlཥkg^-1^ཥmin^-1^ཥrun^-1^, *P* < 0.001) shoes. During overground running, on the other hand, only the 50 mm shoes presented a slope that could be differentiated from zero (-0.36 mlཥkg^-1^ཥmin^-1^ཥrun^-1^, *P* = 0.014), whereas both the 40 mm (-0.14 mlཥkg^-1^ཥmin^-1^ཥrun^-1^, *P* = 0.326) and EL (-0.16 mlཥkg^-1^ཥmin^-1^ཥrun^-1^, *P* = 0.130) shoes had slopes that could not be differentiated from zero.

The results of other physiological variables are shown in Table 2. Of note, HR responses followed a similar profile compared to RE both during treadmill and overground running, showing lower values for the 40 mm shoes compared with the EL shoes, and even lower values for the 50 mm shoes. Minute ventilation differences showed a similar direction but were less prominent, and a statistical difference could only be detected between the 50 mm and EL shoes and between the 40 mm and EL shoes.

### Perceptual Variables

Perceptual responses are listed in Table 3. Whereas both AFT models showed decreased perceived exertion compared with the EL shoes, no difference could be detected between the 50 mm and 40 mm shoes. These results were similar both during treadmill and overground running. The 40 mm shoe had a higher rating of comfort compared with both the EL and 50 mm shoes. When comfort was compared between overground and treadmill running for the same shoe, neither the EL (*P* = 0.592) or 40 mm (*P* = 0.142) showed detectable differences between conditions, whereas comfort was lower for the 50 mm shoes when running overground compared with treadmill running (-0.6 ± 1.2 units, *P* = 0.031). Likewise, when shoes were compared over time (i.e., from visit 2 to visit 3, irrespective of whether on the treadmill or track, using a 2-way repeated measures ANOVA with Holm-Sidak posthoc), only the 50 mm shoes showed an improvement over time (from 5.9 ± 1.8 mm to 6.5 ± 1.9 mm, *P* = 0.042), whereas no differences were detected for the 40 mm (from 7.3 ± 1.3 mm to 7.2 ± 1.6 mm, *P* = 0.755) or EL shoes (from 5.7 ± 2.0 mm to 5.7 ± 2.1 mm, *P* = 0.965).

**Table 3:** Perceptual responses to the different shoes

	Overground			Within Shoe CV (%)		
	40 mm	50 mm	EL	40 mm	50 mm	EL
Impact duration (ms)	67.2 ± 17.7£	66.9 ± 17.4	69.8 ± 19.6	4.4	6.8	5.8
Impact magnitude (g)	4.6 ± 1.5	4.7 ± 1.5	4.5 ± 1.5	3.3	3.8	3.9
Ground contact time (ms)	210.2 ± 14.3	209.7 ± 14.0	209.9 ± 14.8	1.3	1.7	1.1
Cadence (stepsཥmin^-1^)	177.3 ± 7.8	177.2 ± 8.1	177.6 ± 7.9	1.5	1.3	0.9
Duty Factor (%)	37.9 ± 5.0	38.1 ± 5.0	37.9 ± 5.0	2.6	3.8	2.3
Dynamic stability (%)	22.8 ± 4.1	22.4 ± 3.9	22.2 ± 4.0	2.4	2.6	3.6
Vertical Stiffness (kN･m^-1^)	32.5 ± 7.1	32.7 ± 7.1	32.5 ± 7.0			
Leg Stiffness (kN･m^-1^)	10.0 ± 2.2	10.2 ± 2.3	10.0 ± 2.2			

Data are mean ± standard deviation. £ different from EL. 40 mm, AFT shoe with midsole thickness of 40 mm; 50 mm, AFT shoe with midsole thickness of 50 mm; EL, entry-level shoe. The number of symbols denote £*P* < 0.05. CV, coefficient of variation

### Spatiotemporal Variables

Spatiotemporal variables during overground running are displayed in Table 3. The system used for the data collection was not available from the beginning of the experiments (*n* = 2) and technical issues prevented the use of the data from other participants (*n* = 4). As a result, all spatiotemporal values are reported for *n* = 15 participants.

No differences between shoes could be detected between the different shoe conditions during overground running. The only exception was impact duration, which was 2.6 ± 3.3 ms lower for 40 mm compared with EL (~ 3.8%, *P* = 0.024). Although numerically the difference between the 50 mm and EL shoes was larger (2.9 ± 5.5 ms lower for 50 mm vs. EL), no statistical difference could be detected between the two conditions (*P* = 0.107).

## Discussion

This study tested whether an additional centimetre of highly compliant and resilience foam in an AFT shoe already at the upper limit of what is allowed by World Athletics would provide additional improvements in RE. Our results showed that a midsole thickness of 50 mm offered measurable improvements in RE compared with 40 mm, namely of 0.6% during treadmill running and 0.7% during overground running. While the differences in RE were large enough to be reflected in HR, they did not manifest in terms of rating of perceived exertion or spatiotemporal variables. Although differences were consistent across the tested surfaces, no clear relationship in the changes between conditions could be detected. Finally, although the 50 mm shoes ranked best in terms of RE, they also presented the lowest ratings for perceived comfort, which might blunt the transferability of improved RE into performance.

The benchmark shoe used in our study, the Cloudboom Echo 3 (40 mm), displays all the characteristics of an AFT model. Indeed, our test shows that RE was meaningfully improved compared to a traditional running shoe (EL), by nearly four times the 1% that could be expected from the 95 g difference in shoe mass alone [20]. Nonetheless, the 50 mm prototype was still able to improve RE further, albeit by a small amount (0.6–0.7% depending on running surface). It has been argued that higher midsoles would increase frontal plane instability, and that more foam in the midsole could only be helpful to further improve RE if there was not enough foam to begin with, the so-called to a bottom-out phenomenon [6]. This idea was further supported by a later study using AFT shoes, both in terms of no effects on RE as well as increased frontal plane ankle instability [9], the latter finding also confirmed by others showing increased instability at the foot and hip with increased stack heights [21], but not in terms in running coordination or motor variability during running [22]. However, as seen in our data and that of Barrons et al., [9], increasing the midsole thickness also affected the compliance of the shoe, making it softer and absorbing more energy. Thus, more energy could be released even though no “bottom-out” was present in the 40 mm shoe.

It this interesting to note, however, that when comparing the EL and 40 mm shoes, the differences in RE were nearly twice as large during overground running compared with treadmill running, whereas the magnitude of differences between the 50 mm and 40 mm shoes were virtually identical between surfaces. It is well possible that the higher shock absorption and compliance of running treadmills compared with a tartan track offset some of the improvements in RE seen when AFT shoes are worn [23, 24]. Any additional gains provided by the higher midsole in the 50 mm shoes, on the other hand, might be offset by the additional feeling of “instability” reported by the participants and the 50% of turning present during track running.

The disparity between our results and those of Barrons et al. [9] and Bertschy et al. [10] can be due to a range of factors. First and foremost, the effects of shoes on RE cannot be reduced to simply mass and energy return; other factors like plate shape and positioning, shoe geometry and other foam properties likely interact with each other, so that no generalized statement about the effect of midsole thickness on RE can be made from any single study. Second, in the study of Barrons, a single 6-min run for each shoe was used, a design choice that Barrons and other authors later came to call “the fallacy of single trials” [25]. In our study, no differences would be detected if we had limited data collection to a single running bout with each shoe (data not shown), pointing towards a small familiarization effect when running with more unusual shoes, in contrast to what has been recently suggested [26]. A longer familiarization might be needed for the 50 mm shoes, as also evidenced by the negative slope in V̇O_2_ over the time seen only with the 50 mm shoes during overground running. While Bertchy et al. [11], used duplicates of 5-min runs in shoe condition, several other differences between that and the present study could explain the different results, including the use of a firmer EVA-based midsole for the main comparisons and the use of a very extreme 60-mm thick PEBA-based model in a sub-set of five participants, which could have exacerbated shoe instability. Lastly, Bertschy and colleagues matched shoe mass to a rather excessive 465 g from the firm 60-mm shoe condition, which could potentially affect running style. Our study, on the other hand, provides evidence that at very similar speeds as used during the Kona World Championship, unincumbered by additional mass, a 50-mm shoe can indeed provide a measurable (seen even in HR) improvement in RE despite the fact that track running might provide a disadvantage to more unstable shoes due to amount of turning involved. To date, only Barrons and colleagues have investigated the effect of midsole thickness on AFT shoes during sharp curves (radii of 3, 6 and 9 m) [27], reporting no difference between midsole thicknesses of 35 and 50 mm with regards to frontal plane ankle angle and most measures of ground reaction forces, leading the authors to conclude that midsole thickness plays no major role during turning. The track used in the current study had a curve radius of 36 m, so any effects should be further diluted. Considering the effects of curves on running, Taboga and Kram [28] estimated that a 2:01:32 marathon run on a perfect straight line would slow down to 2:01:39 on a 400-m track like the one we used, a difference of 0.08%. Thus, while the existing literature suggests that the effects of the turns in the present study would be minimal, if any, it again should be considered that the geometry of the shoes could play a role at maximizing or minimizing instability.

Despite the differences in RE measure between shoes, no differences in spatiotemporal variables were detected between the 40 mm and 50 mm shoes, in line with the findings of Barrons et al., [9] and Bertschy et al. [10], except for a small decrease in impact duration with the 40 mm shoes compared with EL (and numerically for the 50 mm vs. EL). While the magnitude of these differences was too small to draw any meaningful conclusions, these findings lend support to a higher rate of loading of the lower limbs when running with AFT models, which could have implications for the incidence of injuries. We also did not detect changes in vertical or leg stiffness with increasing AFT midsole thickness, again in line with Bertschy and colleagues when comparing leg stiffness but not vertical stiffness. Looking into the determinants of vertical stiffness, neither ground contact time nor flight time changed in either study as a function of increased midsole thickness, so differences should reside in the determined changes in centre of mass oscillation. This was measured in the study with motion capture of Bertschy and colleagues, but instead calculated following a modelled approach in our study, which could have hidden small changes in values caused by deviations from the ideal model.

Even though the differences in RE between shoes were consistent during both treadmill and overground running, we could not detect a relationship between the differences in V̇O_2_ measured on both conditions, likely due to the very small difference between models. This finding is not unique, as others have also found opposite effects between aspects of footwear construction when tested during treadmill or overground running [29], although the latter study used running along a 15-m runway as a model for overground running. While these findings to not invalidate treadmill-based testing, they should caution athletes from choosing footwear based exclusively on minute RE differences between shoe models, which might not reproducible in other surfaces or even between days under similar conditions [9]. Although our results might also be specific to the treadmill used in our study, the present data does suggest that athletes should be encouraged to test their footwear during overground running, particularly during asphalt running if this is the surface relevant for competition. Importantly, while it could be argued that the different ergospirometers used during treadmill and overground running would be responsible for the lack of correlations, these should lead to a systematic difference between the tested surfaces, where one device consistently over- or underreads the other, thus affecting the intercept of a correlation, but not its slope or strength. Our two devices have excellent and similar repeatability [30], which should have sufficed to measure a relationship between conditions, if one was present.

Despite the 0.7% difference in RE, perceived exertion was not lower with the 50 mm compared with the 40 mm shoes, likely because the differences in RE are too small to be detected by the scales used. Nonetheless, small differences in RE between shoe models have been shown to yield performance differences [31], which might be independent of conscious perception. Furthermore, although we could not find meaningful correlations between perceived comfort and exertion or RE, as previously suggested [32], it is tempting to speculate that the lower comfort perceived with the 50 mm shoes could have offset potential effects of improved RE on perceived effort. As the 50 mm shoes were (anecdotally) reported by the participants as “feeling unstable”, It could be that this perception might have led to a greater focus on physical sensations during the run.

### Limitations

This study was not without its limitations. We used two different ergospirometers, one for the laboratory condition and another for running outdoor. We did not, however, compare the two conditions directly, but instead compared shoes within day, measured using the same system and calibration settings.

The overground running trials were performed outdoors, when environmental conditions could not be controlled. To this end, rainy or extreme weather days were avoided, and again all comparisons were made within the testing day for each participant (i.e., within 2 h, without meaningful differences in weather throughout the sessions).

Technical difficulties resulted in a smaller sample size for the spatiotemporal data. This is problematic as these variables exhibit slightly greater variability compared with physiological data such as V̇O_2_, thus decreasing our statistical power. Thus, the lack of differences in spatiotemporal variables found in our study should not be seen as strong evidence in favour of similarity between the different shoes tested.

We did not have access to a wide range of shoe sizes, as the 50 mm only existed in three different sizes. While we did not exclude women from participation, we are aware that the population of women able to run at 16 kmཥh^-1^ and having shoe sizes US men 9.0–11.0 is very small. Although it would have been ideal to have a more balanced sex distribution, to date research on AFT does not seem to suggest the presence of strong sex-dependent effects.

Lastly, while our study design does not allow us to separate the effect of midsole thickness from the changes in mechanical properties of the shoes that come along with it, we view these as a reflection of the real-world impact of changing the height of the shoe with the same material as the standard midsole. For the same reason, we chose not to equate the mass of the 40 mm and 50 mm shoes, as done by others, as it is unrealistic to expect that the midsole thickness could be increased without any added mass to the shoe. In any case, the added mass of the 50 mm shoes acted against any possible benefit to RE, meaning that the differences elicited by the higher midsole had to first overcome the disadvantage of the added mass to then provide an advantage in RE, as originally described by Frederick [20] and recently reviewed by Ruiz-Alias et al. [33].

## Conclusions

The present study shows that contrary to recent evidence, increasing midsole thickness beyond current regulations can further improve RE, albeit to a small extent. It remains to be seen whether such small increments would translate into performance gains. Our data does suggest, however, that the 40-mm upper limit for midsole thickness imposed by World Athletics for road running events does not represent a ceiling beyond which further increases become self-limiting. Given that the current rules might discourage manufacturers from venturing outside the 40-mm upper limit, whether the present study represents evidence in favour of the imposed restrictions or whether it shows that current rules prevent further innovation is open for debate.

## Supplementary Information

Below is the link to the electronic supplementary material.

Supplementary Material 1

## Data Availability

The datasets used and/or analysed during the current study are available from the corresponding author on reasonable request. Competing interests. The authors declare that they have no competing interests.

## Abbreviations

AFT: advanced footwear technology
DXA: dual x-ray densitometry
EL: entry-level shoe
EVA: ethyl vinyl acetate
PEBA: polyether block amide
RE: running economy
SD: standard deviation
VAS: visual analogue scale
40mm AFT: shoe with midsole thickness of 40 mm
50mm AFT: shoe with midsole thickness of 50 mm
