# Supplementary material for: The Effect of Midsole Thickness on Running Economy, Spatiotemporal Values and Perceptions of Comfort and Exertion in Well-trained Runners: A Randomized, Cross-over Trial
Source: Sports Med Open. 2025 Oct 1;11:108. doi: 10.1186/s40798-025-00911-z (PMC12488538; doi:10.1186/s40798-025-00911-z)
Supplement: Supplementary file 1 — Supplementary Material 1 [file 40798_2025_911_MOESM1_ESM.pdf]

Supplementary Information

Journal: Sports Medicine – Open

Title: The effect of midsole thickness on running economy, spatiotemporal values and perceptions of comfort and exertion in well-trained runners: a randomized, cross-over trial.

Authors: Gian-Andri Baumann [a], Kai Biedermann [a], Enea Item [a], Christina M. Spengler [a], Fernando G. Beltrami [a]

[a] Exercise Physiology Lab, Institute of Human Movement Sciences and Sport, ETH Zurich, Zurich, Switzerland

Relative $\dot{V}O_2$ – Treadmill (in ml $O_2 \cdot kg^{-1} \cdot min^{-1}$)

Participant	40mm				50mm				EL			
	Run 1	Run 2	Run 3	Run 4	Run 1	Run 2	Run 3	Run 4	Run 1	Run 2	Run 3	Run 4
1	64.9	63.6	64.7		67.9	62.3	65.1		67.6	61.3	66.0	
2	61.9	62.7	57.9	60.3	62.5	62.0	57.7	60.0	64.7	65.2	61.1	60.9
3	54.8	52.7	52.6	52.4	53.9	53.3	52.6	52.3	58.4	52.1	53.2	53.2
4	60.5	59.4	61.2	61.3	60.9	58.8	60.7	60.9	62.0	60.4	61.7	61.7
5	59.6	58.5	57.5	55.7	58.1	59.0	57.1	55.6	60.7	60.5	57.4	57.0
6	56.9	54.9	55.8	54.3	56.0	55.5	54.2	53.9	58.1	54.9	55.8	55.8
7	54.7	54.5	54.2	54.3	54.6	54.1	53.8	56.6	54.1	56.4	56.5	55.0
8	60.5	59.9	58.0	61.0	58.6	57.0	57.5	59.7	62.3	62.7	62.6	61.0
9	59.6	59.7	56.7	56.9	60.4	58.2	57.4	56.1	61.4	59.7	59.0	55.7
10	59.1	55.2	53.7	52.9	56.2	55.0	53.7	52.8	58.9	57.0	55.6	55.9
11	59.6	58.7	57.0	59.7	59.6	59.5	57.5	58.9	60.8	60.8	59.5	61.5
12	59.6	58.5	58.3	57.6	57.3	59.4	58.5	59.5	60.4	60.7	59.8	59.8
13	54.8	54.2	53.7	51.5	55.7	52.7	53.9	51.0	54.7	53.5	55.0	53.8
14	63.4	62.9	62.3	62.0	62.6	61.8	61.7	62.5	65.4	64.4	63.9	62.6
15	49.2	49.2	47.5	48.0	51.8	49.6	49.8	47.6	52.9	49.6	47.9	50.8
16	53.0	52.2	53.6	56.1	53.9	51.3	53.1	52.0	55.7	53.7	54.9	55.4

17	51.6	52.4	52.5	50.5	51.4	52.4	50.8	50.6	54.8	53.7	53.2	52.2
18	53.5	52.7	52.2	52.9	51.9	52.8	52.2	51.3	52.2	55.4	53.6	53.1
19	49.2	48.6	49.3	48.4	49.3	47.8	49.2	48.7	51.8	51.2	51.0	50.7
20	52.5	51.7	53.0	53.4	50.8	51.2	51.5	51.5	53.3	55.5	54.2	54.1
21	55.0	54.8	54.9	53.4	53.4	54.9	54.4	53.4	56.4	56.4	55.9	55.5

Note that due to technical difficulties Run 4 for participant 1 is not available.

Relative $\dot{V}O_2$ – Overground (in ml $O_2 \cdot kg^{-1} \cdot min^{-1}$)

Participant	40mm				50mm				EL			
	Run 1	Run 2	Run 3	Run 4	Run 1	Run 2	Run 3	Run 4	Run 1	Run 2	Run 3	Run 4
1	57.7	55.4	55.7	55.4	54.9	55.8	54.9	55.1	58.1	58.0	57.9	57.1
2	51.6	52.8	51.1	52.1	51.7	52.8	52.9	51.8	55.9	54.8	57.0	54.9
3	53.8	54.5	53.6	54.7	54.7	54.9	52.9	53.7	57.4	55.6	55.5	55.3
4	51.9	50.6	52.5	50.8	50.3	51.2	51.6	51.0	53.2	53.5	55.5	54.1
5	50.0	51.1	52.8	49.9	49.8	49.9	47.5	49.0	52.7	54.4	52.7	52.5
6	50.0	49.9	48.4	49.1	50.4	49.2	50.1	47.8	51.4	52.4	52.0	50.0
7	50.6	51.8	50.1	50.2	51.2	50.9	51.4	48.9	53.5	50.5	51.1	50.2
8	52.9	54.1	55.0	54.7	54.1	53.8	52.8	52.2	58.1	55.7	56.6	56.9
9	52.1	52.4	52.3	51.5	51.2	51.2	50.6	50.5	54.3	53.7	52.8	53.5
10	47.2	48.5	47.2	46.9	47.9	47.6	46.9	48.4	50.6	49.5	50.3	50.7
11	54.3	52.8	54.7	55.2	54.4	54.6	53.1	52.4	55.9	56.6	56.5	55.9
12	50.9	51.2	50.5	50.3	51.6	50.6	49.7	52.3	53.3	52.9	53.1	51.6
13	47.1	46.9	47.7	46.5	46.3	47.2	48.0	47.3	47.9	48.4	49.0	46.7
14	49.0	47.1	50.6	47.1	48.6	46.8	49.9	46.5	51.6	50.5	48.6	49.6
15	45.9	48.0	45.4	45.8	46.2	46.1	43.9	44.3	48.6	48.0	47.7	47.8
16	50.7	49.8	49.4	52.1	52.1	49.7	51.3	49.5	52.7	52.5	52.8	54.3

17	49.9	49.7	50.4	49.9	50.7	51.0	50.4	50.7	52.9	49.4	52.9	53.0
18	47.4	52.1	51.9	50.5	49.5	49.3	49.8	50.6	53.8	53.1	52.8	52.7
19	54.1	52.9	49.1	48.9	54.1	50.5	47.6	48.9	53.0	53.1	53.8	51.5
20	49.2	47.7	50.5	49.0	47.9	48.1	47.9	49.7	47.7	51.1	50.6	51.7
21	54.4	54.6	51.9	50.7	54.3	55.3	53.6	50.2	55.0	56.1	53.2	55.3

Metabolic Power – Treadmill (in W・kg^{-1})

Participant	40mm				50mm				EL			
	Run 1	Run 2	Run 3	Run 4	Run 1	Run 2	Run 3	Run 4	Run 1	Run 2	Run 3	Run 4
1	22.6	22.1	22.4		23.8	21.6	22.5		23.7	21.5	22.9	
2	21.7	21.9	20.4	21.1	22.1	21.6	20.2	20.8	22.6	23.0	21.5	21.5
3	18.9	18.2	18.2	18.1	18.7	18.5	18.2	18.1	20.3	18.0	18.4	18.4
4	20.9	20.5	21.0	21.0	21.0	20.3	20.9	20.9	21.6	20.9	21.3	21.2
5	20.7	20.3	20.0	19.2	20.1	20.4	19.8	19.2	21.1	21.0	19.9	19.8
6	19.7	19.0	19.3	18.8	19.5	19.3	18.8	18.7	20.2	19.2	19.4	19.4
7	19.0	18.9	18.8	18.8	19.0	18.8	18.7	19.6	18.9	19.6	19.6	19.1
8	21.0	20.8	20.1	21.1	20.4	19.7	19.9	20.6	21.7	21.8	21.8	21.1
9	20.7	20.7	19.6	19.7	20.9	20.2	19.8	19.4	21.4	20.7	20.5	19.4
10	20.5	19.1	18.5	18.2	19.4	19.0	18.5	18.2	20.4	19.7	19.2	19.3
11	20.9	20.5	19.9	20.8	20.8	20.8	20.1	20.5	21.4	21.3	20.8	21.6
12	20.6	20.1	20.1	19.9	19.8	20.4	20.2	20.5	20.9	20.9	20.7	20.6
13	18.9	18.7	18.5	17.8	19.2	18.2	18.6	17.6	18.9	18.5	19.0	18.6
14	21.8	21.6	21.3	21.2	21.5	21.2	21.2	21.4	22.6	22.2	22.0	21.5
15	17.1	17.1	16.5	16.7	18.0	17.3	17.4	16.6	18.4	17.4	16.8	17.8
16	18.6	18.3	18.8	19.6	18.9	17.9	18.6	18.2	19.6	18.9	19.3	19.5

17	18.0	18.3	18.3	17.6	17.9	18.2	17.8	17.6	19.2	18.8	18.6	18.3
18	18.7	18.4	18.2	18.4	18.1	18.4	18.1	17.8	18.2	19.4	18.8	18.5
19	17.2	17.0	17.2	16.8	17.2	16.6	17.1	16.9	18.1	17.9	17.8	17.7
20	18.3	18.0	18.4	18.5	17.7	17.8	17.8	17.9	18.6	19.3	18.9	18.8
21	19.2	19.1	19.1	18.6	18.7	19.1	19.0	18.6	19.7	19.7	19.5	19.4

Metabolic Power - Overground (in W·kg^{-1})

Participant	40mm				50mm				EL			
	Run 1	Run 2	Run 3	Run 4	Run 1	Run 2	Run 3	Run 4	Run 1	Run 2	Run 3	Run 4
1	20.0	19.2	19.2	19.2	19.0	19.3	18.9	19.0	20.2	20.1	20.1	19.8
2	18.0	18.4	17.8	18.1	18.1	18.4	18.4	18.0	19.5	19.1	19.9	19.1
3	18.9	19.1	18.8	19.1	19.2	19.2	18.5	18.8	20.3	19.5	19.5	19.4
4	18.1	17.6	18.2	17.6	17.5	17.8	17.9	17.7	18.6	18.6	19.3	18.7
5	17.5	17.8	18.4	17.3	17.4	17.3	16.5	17.0	18.4	19.0	18.4	18.3
6	17.3	17.2	16.7	16.9	17.4	17.0	17.3	16.5	17.8	18.2	18.0	17.3
7	17.6	18.1	17.4	17.4	17.9	17.7	17.9	17.0	18.7	17.6	17.8	17.4
8	18.3	18.8	19.1	19.0	18.8	18.7	18.3	18.1	20.2	19.3	19.6	19.7
9	18.2	18.3	18.2	18.0	17.8	17.9	17.7	17.6	19.0	18.8	18.4	18.7
10	16.4	16.8	16.4	16.3	16.6	16.5	16.3	16.8	17.7	17.2	17.5	17.6
11	19.1	18.4	19.1	19.3	19.0	19.1	18.5	18.3	19.6	19.8	19.8	19.6
12	17.7	17.8	17.5	17.5	18.0	17.6	17.3	18.1	18.6	18.4	18.5	17.9
13	16.5	16.3	16.7	16.2	16.2	16.5	16.8	16.5	16.8	16.9	17.1	16.3
14	17.0	16.4	17.6	16.4	16.9	16.3	17.1	16.1	18.1	17.6	16.9	17.3
15	15.9	16.7	15.7	15.8	16.0	15.9	15.1	15.2	16.9	16.7	16.6	16.4
16	17.8	17.4	17.3	18.2	18.2	17.4	17.9	17.3	18.6	18.5	18.6	19.1

17	17.4	17.3	17.5	17.4	17.7	17.8	17.5	17.7	18.5	17.3	18.4	18.5
18	16.5	18.2	18.1	17.6	17.2	17.2	17.3	17.6	18.8	18.5	18.4	18.3
19	18.8	18.4	17.0	17.0	18.8	17.5	16.5	16.9	18.4	18.5	18.7	17.9
20	17.1	16.5	17.5	17.0	16.6	16.7	16.6	17.2	16.6	17.8	17.6	18.0
21	18.9	19.0	18.0	17.6	19.0	19.3	18.6	17.4	19.1	19.6	18.5	19.2
